# Higher Platelet-to-Lymphocyte Ratio Is Associated With Worse Outcomes After Intravenous Thrombolysis in Acute Ischaemic Stroke

**DOI:** 10.3389/fneur.2019.01192

**Published:** 2019-11-13

**Authors:** Jing-Han Xu, Xin-Wei He, Qiang Li, Jian-Ren Liu, Mei-Ting Zhuang, Fei-Fei Huang, Guan-Shui Bao

**Affiliations:** ^1^Department of Neurology, Shanghai Ninth People's Hospital, Shanghai Jiao Tong University School of Medicine, Shanghai, China; ^2^Clinical Research Center, Shanghai Jiao Tong University School of Medicine, Shanghai, China

**Keywords:** acute ischaemic stroke, intravenous thrombolysis, platelet-to-lymphocyte ratio, stroke severity, prognosis

## Abstract

**Objective:** The platelet-to-lymphocyte ratio (PLR) is a new marker of atherosclerotic inflammation and has been identified as a predictive factor in cardiovascular diseases, but its significance in patients with acute ischaemic stroke (AIS) who have undergone intravenous thrombolysis (IVT) is still unknown.

**Methods:** Consecutive patients who were treated with IVT using recombinant tissue plasminogen activator (rtPA) for AIS were included from May 2012 to August 2018. The PLR was calculated according to platelet and lymphocyte counts within 24 h after thrombolysis therapy. Functional outcomes were assessed by the modified Rankin Scale (mRS) at 3 months after thrombolysis. Stroke severity was assessed by National Institutes of Health Stroke Scale (NIHSS) scores. The primary endpoint was an unfavorable outcome (mRS > 2), and the secondary endpoint was death at 3 months.

**Results:** A total of 286 patients were included in the study. The median age was 69.5 (59.0–80.0) years, and 59.1% of patients were men. A total of 120 (42.0%) patients had an unfavorable outcome, and 38 (13.2%) died. Patients with an unfavorable outcome had significantly higher PLR values compared with those with a favorable outcome [172.5 (105.3–239.0) vs. 139 (97.0–194.5), *P* = 0.008], and the PLR values of the patients who died at 3 months were higher than those of the surviving patients [189.5 (127.5–289.0) vs. 142.0 (98.0–215.5), *P* = 0.006]. After adjustment for other variables, the PLR was independently associated with the two endpoints: unfavorable outcome (OR 2.220, 95% CI 1.245–3.957, *P* = 0.007) and death (OR 2.825, 95% CI 1.050–7.601, *P* = 0.040) at 3 months after thrombolysis. In addition, PLR was correlated with the NIHSS score (R = 0.230, *P* < 0.001).

**Conclusions:** Higher PLR levels were independently associated with an unfavorable outcome and death at 3 months in AIS patients treated with IVT.

## Introduction

Worldwide, stroke is the leading cause of death and disability among adults and has conferred substantial economic and social burdens (1). Acute ischaemic stroke (AIS) is the most common type of stroke in China (2). In AIS management, despite its curative treatment expanding from intravenous thrombolysis (IVT) to arterial thrombolysis and thrombectomy, IVT using rtPA is still the first recommended treatment to restore blood flow and has been promoted and gradually popularized in many basic-level hospitals in recent years (3–5). However, after recanalization, patients may have complications, such as vascular reocclusion, brain oedema, and intracerebral hemorrhage, that affect the functional outcome (6).

Blood cell testing is necessary and routine for AIS patients treated with IVT. The platelet-to-lymphocyte ratio (PLR) is a new, affordable, available and composite biomarker of the inflammation in cerebrovascular disease that combines the prognostic value of single platelet and lymphocyte counts in the field of stroke. PLR has at least two advantages. One is that it is a comprehensive indicator that may contribute additional information to traditional markers. The other advantage is that it is a ratio and is thus more stable than a single blood parameter that can vary because of multiple factors such as over-hydration, dehydration, and treatment of blood specimens (7).

Recently, these advantages of PLR have been confirmed in a variety of diseases, including myocardial infarction, cerebral infarction and peripheral ischaemia (8–10). In addition, studies have suggested a potential association of high PLR levels with the severity of coronary atherosclerosis and symptomatic internal carotid artery stenosis (11, 12). Recent studies showed that high PLR values increased the size of the infarcted area and the incidence of poor prognosis in AIS patients (13). However, IVT using rtPA influence peripheral blood platelet counts and lymphocyte concentrations, resulting in changes in the value of the PLR (14). Whether a similar association exists in AIS patients treated with IVT has not yet been reported.

In this study, for the first time, we therefore aimed to analyse whether the PLR was associated with 3-month prognosis (including functional outcome and death) of AIS patients treated with IVT.

## Materials and Methods

### Study Population

This retrospective study based on a prospective database included consecutive patients with AIS who received IVT treatment using rtPA at the Shanghai Ninth People's Hospital, Shanghai Jiao Tong University School of Medicine.

Treatment principles of our hospital strictly followed the Guidelines for the early management of patients with acute ischemic stroke from the American Heart Association/American Stroke Association (4, 5, 15, 16). A cranial computerized tomography (CT) scan was performed before IVT to rule out the possibility of haemorrhagic stroke on arrival at the emergency department. Clinical guideline recommendations for the time window (from symptom onset to IVT) extended from 3.0 to 4.5 h, and rtPA was infused (i.e., 0.9 mg/kg, maximum 90 mg, 10% of the dose as a bolus over 1 min and the remainder as a 60-min intravenous infusion). Another cranial CT or magnetic resonance imaging (MRI) scan was performed 24 h after the therapy in cases of clinical worsening or when the symptoms changed. Other examinations and treatments were performed according to the AIS guidelines. Stroke etiology was determined according to the criteria of the Trial of Org 10172 in Acute Stroke Treatment (TOAST) (17).

**Figure 1:**
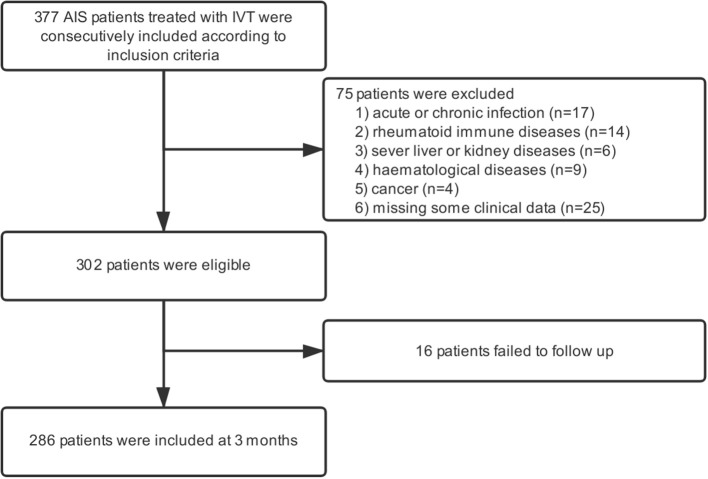
Study flow chart. AIS, acute ischaemic stroke; IVT, intravenous thrombolysis.

In the present study, 377 patients received intravenous thrombolysis at the stroke center of Shanghai Ninth People's Hospital from May 2012 to August 2018, and all of them were included in the study. Moreover, we excluded patients with acute or chronic infection, rheumatoid immune diseases, and other prior systemic diseases, including severe liver and kidney diseases, hematological systemic diseases, cancer, etc. In addition, patients who failed to follow up at 3 months were also regarded as ineligible (Figure 1).

The experimental study was approved by the Ethics Committee of Shanghai Ninth People's Hospital, and written informed consent was obtained from the patients or their relatives. All procedures were carried out in accordance with the code of ethics of the 1975 Declaration of Helsinki.

### Data Collection and Definition

Demographic characteristics (such as age and sex), baseline vital signs (systolic blood pressure and diastolic blood pressure), baseline vascular risk factors (smoking, alcohol abuse, history of hypertension, dyslipidaemia, diabetes mellitus, history of stroke, coronary artery disease, and atrial fibrillation) and characteristics of the procedure (onset-to-treatment time, bridging endovascular therapy) were reviewed from our medical institutional database. Blood sampling for laboratory tests was limited to within 24 h after the onset of symptoms. Laboratory and imaging information were obtained from an automatic testing system. Following the measurement, the PLR was calculated by the platelet count/lymphocyte count formula.

Risk factors were evaluated as follows: hypertension is defined as repeated systolic blood pressure/diastolic blood pressure ≥140/90 mmHg, a history of previous hypertension or taking antihypertensive drugs. Diabetes is defined as a history of previous diabetes or by the use of diabetes medications, or more than two measurements of fasting plasma glucose >7.0 mmol/L or random plasma glucose >11.1 mmol/L. Coronary heart disease (CHD) is defined as a history of previous CHD or a CHD attack at the time. Atrial fibrillation (AF) is defined as any previous known AF episode or electrocardiogram of AF recorded during the hospital. Smoker is defined as smoking until the symptom onset of stroke or quit smoking within 1 year, and drinker is defined as alcohol abuse >2 U/d.

### Study Endpoints

Stroke severity was assessed according to the national institutes of health stroke score (NIHSS) (18), and the evaluation was performed by a neurologist in the emergency department. Moderate-to-severe stroke was defined as NIHSS scores ≥8 before IVT, consistent with the existing literature (19, 20). The follow-up time was 3 months after AIS, and it was performed by specialist nurses by telephone. The primary endpoint was evaluated by the modified Rankin Scale (mRS); a favorable outcome was defined as mRS ≤ 2, while an unfavorable outcome was defined as mRS > 2 (21). The secondary endpoint was the occurrence of death associated with the stroke episode.

### Statistical Analysis

Data were processed using SPSS 22.0 (IBM, Chicago, IL, USA). Distribution normality was tested by the Kolmogorov–Smirnov test (Supplementary Table S1). Continuous variables were exhibited by mean ± standard deviation (SD) or medians and interquartile range (IQR), which were analyzed by independent test or Mann–Whitney *U*-test, respectively. Categorical data were represented as frequencies (percentages) and were analyzed using the Chi-Square test or Fisher's exact test. Correlation between PLR and any other data was assessed using the Spearman test. The cut-off point of PLR was calculated according to the receiver operating characteristic (ROC) curve, and the PLR level was dichotomized at high (i.e., at the cut-off value or above) or low (i.e., below the cut-off) values while involved in the multivariate analysis. Multivariate analysis was performed using logistic regression models, adjusted for other variables selected from univariate analyses. A *P* < 0.05 was considered significant for all analyses.

## Results

### Baseline Characteristics of the Patients

In our study, a total of 377 patients treated with IVT were included according to inclusion criteria. After applying the exclusion criteria, 75 patients were excluded from the study. Additionally, 16 patients failed to follow up 3 months after AIS (Figure 1). As a result, 286 patients were eligible for the study, which consisted of 169 (59.1%) males and a median age of 69.5 (59.0–80.0) years. The NIHSS scores before thrombolysis were 8 (3–13). The symptom onset to needle time was 160 (114–210) min. In addition, 44 patients received bridging endovascular therapy after IVT. The baseline demographic and clinical characteristics of the patients are provided in Table 1. According to the TOAST criteria, 35.3% of patients were large-artery atherosclerosis (LAA), 20.3% were cardioembolism (CE), 26.2% were small-vessel occlusion (SVO), and 18.2% were other determined/undetermined etiologies.

**Table 1:** Baseline characteristics of patients according to 3-month study endpoints.

	**Total(*n* = 286)**	**Favorable(*n* = 166)**	**Unfavorable(*n* = 120)**	***P*-value**	**Alive(*n* = 248)**	**Dead(*n* = 38)**	***P*-value**
**Demographic data**							
Age, years	69.5 (59.0–80.0)	64.0 (57.0–76.0)	79.0 (66.0–83.0)	<0.001	68.0 (59.0–79.0)	79.0 (66.0–83.0)	0.001
Male, n (%)	169 (59.1)	102 (61.4)	102 (61.4)	0.341	151 (60.9)	18 (47.4)	0.114
**Stroke risk factors, n (%)**							
Hypertension	204 (71.3)	123 (74.1)	81 (67.5)	0.223	178 (71.8)	26 (68.4)	0.670
Diabetes mellitus	83 (29)	40 (24.1)	43 (35.8)	0.031	68 (27.4)	68 (27.4)	0.127
History of stroke	68 (23.8)	28 (16.9)	40 (33.3)	0.001	52 (21.0)	16 (42.1)	0.004
AF	91 (31.8)	47 (28.3)	44 (36.7)	0.134	73 (29.4)	18 (47.4)	0.027
CHD	76 (26.6)	41 (24.7)	35 (29.2)	0.399	58 (23.4)	18 (47.4)	0.002
Smoking	180 (51.1)	92 (55.4)	88 (73.3)	0.002	149 (60.1)	31 (81.6)	0.011
Alcohol abuse	52 (18.2)	26 (15.7)	26 (21.7)	0.194	43 (17.3)	9 (23.7)	0.345
**Vital signs**							
SBP, mmHg	150 (135–170)	150 (135–165)	150 (135–170)	0.492	150 (135–169)	150 (136–170)	0.760
DBP, mmHg	81 (75–90)	83 (77–90)	80 (75–90)	0.538	81 (76–90)	80 (73–90)	0.271
**Laboratory data**							
TG, mmol/L	1.24 (0.92–1.79)	1.37 (0.92–1.98)	1.14 (0.93–1.67)	0.155	1.28 (0.91–1.87)	1.09 (0.95–1.44)	0.200
TC, mmol/L	4.38 ± 1.09	4.34 ± 1.03	4.38 ± 1.20	0.738	4.35 ± 1.04	4.38 ± 1.48	0.915
LDL, mmol/L	2.91 ± 0.93	2.95 ± 0.94	2.86 ± 0.91	0.449	2.92 ± 0.92	2.85 ± 1.00	0.709
HDL, mmol/L	1.04 (0.86–1.24)	1.03 (0.83–1.23)	1.10 (0.89–1.28)	0.068	1.05 (0.87–1.23)	1.17 (0.85–1.37)	0.327
HCY, μmol/L	12.40 (9.50–16.60)	11.80 (9.15–16.25)	13.10 (9.75–18.20)	0.065	13.00 (10.00–17.00)	12.00 (10.00–15.00)	0.392
Platelet, 10^3^/μl	186.0 (152.0–224.0)	193.0 (156.5–225.3)	176.0 (142.8–215.5)	0.075	190.0 (153.0–224.0)	180.0 (151.0–224.0)	0.643
Lymphocyte, 10^3^/μl	1.3 (0–1.8)	1.5(1.0–1.9)	1.1 (0.7–1.6)	0.001	1.4 (0.9–1.9)	1.0 (0.7–1.4)	0.001
PLR	145.0 (100.5–219.5)	139.0 (97.0–194.5)	172.5 (105.3–239.0)	0.008	142.0 (98.0–215.5)	189.5 (127.5–289.0)	0.006
**Stroke evaluation**							
NIHSS before IVT, points	8 (3-15)	5 (2-10)	13 (7-18)	<0.001	7 (3-13)	16 (8-19)	<0.001
Time to needle, min	160 (114–210)	159 (120–208)	165 (110–214)	0.708	158 (110–207)	180 (139–230)	0.085
Endovascular therapy, n (%)	44 (15.4)	13 (7.8)	31 (25.8)	<0.001	33 (13.3)	11 (28.9)	0.013
**TOAST subtype, n (%)**		<0.001		0.003
Large-artery atherosclerosis	101 (35.3)	64 (38.6)	37 (30.8)		85 (34.3)	16 (42.1)	
Cardioembo-lim	58 (20.3)	46 (27.7)	12 (10.0)		58 (23.4)	0 (0.0)	
Small-vessel occlusion	75 (26.2)	28 (16.9)	47 (39.2)		59 (23.8)	16 (42.1)	
Other determined/undetermined	52 (18.2)	28 (16.9)	24 (20.2)		46 (18.5)	6 (15.8)	

*Values are presented as medians (interquartile range) for continuous variables and numbers (percentages) for categorical variables*.

*AF, atrial fibrillation; CHD, coronary heart disease; SBP, systolic blood pressure; DBP, diastolic blood pressure; TG, triglycerides; TC, total cholesterol; LDL, low-density lipoproteins; HDL, high-density lipoproteins; HCY, homocysteine; PLR, platelet-to-lymphocyte ratio; NIHSS, National Institutes of Health Stroke Scale; IVT, intravenous thrombolysis*.

Exploring the links between the PLR and the severity of stroke, a significant correlation was detected between the NHISS score before thrombolysis (rho = 0.230, *P* < 0.001). According to the NIHSS score, there were 154 cases with a moderate-to-severe stroke and 132 cases with a mild stroke. The PLR level was higher in the moderate-to-severe group than in the mild group [166.0 (108.5–240.0) vs. 129.0 (93.5–210.3); *P* = 0.001; Table 2].

**Table 2:** PLR stratified by stroke severity and 3-month study endpoints.

**Characteristics**	**PLR**				
	**Yes**	**No**	***P***	**Adjusted OR** **(95% CI)**	***P***
M-to-S stroke	166.0 (108.5–240.0)	129.0 (93.5–210.3)	0.001	/	/
Unfavorable	172.5 (105.3–239.0)	139.0 (97.0–194.5)	0.008	2.220 (1.245–3.957)	0.007
Death	189.5 (127.5–289.0)	142.0 (98.0–215.5)	0.006	2.825 (1.050–7.601)	0.004

*Values are presented as medians (IQRs). Moderate-to-severe stroke was defined as an NIHSS score ≥8 before intravenous thrombolysis*.

*PLR, platelet-to-lymphocyte ratio; M-to-S, moderate-to-severe; OR, odds ratio; CI, confidence interval*.

### The Association of Higher PLR Levels With an Unfavorable Outcome

All participants were divided into two subgroups according to main endpoint, and the baseline characteristics and outcomes of the study population are detailed in Table 1. Among 286 patients, 166 (58.0%) presented a favorable outcome, and 120 (42.0%) presented an unfavorable outcome at 3 months.

In the univariate analysis, patients with the favorable and unfavorable prognosis were similar in most characteristics except for age, diabetes mellitus, history of stroke, smoking, NIHSS scores before thrombolysis and endovascular therapy (Table 1).

**Figure 2:**
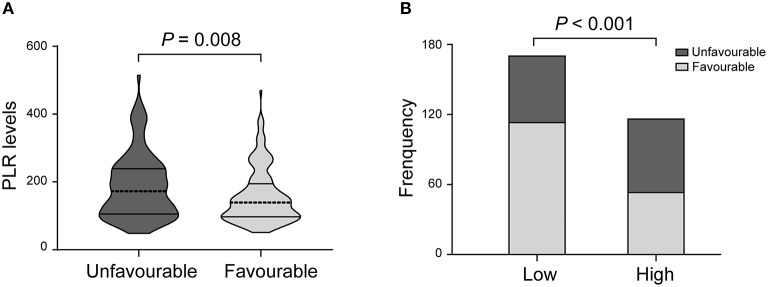
PLR levels and functional outcome. **(A)** Violin-plot graph of PLR distribution in patients with favorable and unfavorable prognosis. **(B)** Comparison of outcomes between patients at high (i.e., at the cut-off value or above) and low (i.e., below the cut-off) levels for platelet-to-lymphocyte ratio (PLR).

There was a statistically significant difference in PLR levels between the favorable and unfavorable outcomes [139.0 (97.0–194.5) vs. 172.5 (105.3–239.0); *P* = 0.008; Table 2, Figure 2A]. In addition, a cut-off value of PLR was obtained according to the ROC curve, and an unadjusted comparison of the 2 groups of patients divided by the cut-off value of PLR showed less favorable outcome in the higher group for PLR (*P* < 0.001, Figure 2B).

After adjusting for confounders, the PLR level remained an autocephaly predictor of unfavorable outcome in AIS patients treated with IVT (AOR 2.220, 95% CI, 1.245–3.957, *P* = 0.007; Table 2). In addition, we found that age, NIHSS score and history of stroke were also predictors of an unfavorable outcome.

### The Association of Higher PLR Levels With the Occurrence of Death

Three months after their strokes, 38 patients (13.3%) died, and 248 (86.7%) were still alive (Table 1). Compared with the patients who survived, the deceased patients were more likely to have cardiovascular risk factors, frequent smoking, a history of stroke, a higher age, higher NIHSS scores and a higher level of PLR. There was no marked difference among all participants in other laboratory analyses.

**Figure 3:**
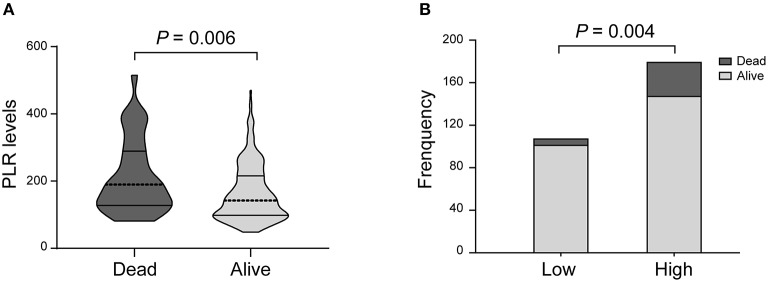
PLR levels and occurrence of death. **(A)** Violin-plot graph of PLR distribution in deceased and surviving patients. **(B)** Comparison of outcomes between patients at high (i.e., at the cut-off value or above) and low (i.e., below the cut-off) levels for platelet-to-lymphocyte ratio (PLR).

In the univariate analysis, the PLR values in the patients who died at 3 months were higher than those in the surviving patients [189.5 (127.5–289.0) vs. 142.0 (98.0–215.5); *P* = 0.006; Table 2, Figure 3A]. A cut-off value of PLR was obtained from the ROC curve, and an unadjusted comparison of the 2 groups of patients divided by the cut-off value of PLR showed higher mortality rates in the group with higher PLR (*P* = 0.003; Figure 3B).

A multiple logistic regression analysis showed that higher PLR levels were independently predictive of death after treatment with IVT (AOR, 2.825, 95% CI, 1.050–7.601, *P* = 0.004; Table 2). In addition, we found that NIHSS score and history of stroke were also valuable predictors of death.

## Discussion

The study first investigated the clinical significance of the PLR on 3-month functional outcomes and death in AIS patients treated with IVT. The results showed that high PLR levels were associated with a short-term unfavorable outcome and death, suggesting that a higher PLR level might be a risk predictor of 90-day prognosis in AIS patients treated with IVT. Our study also found that PLR levels were related to the severity of stroke in AIS patients.

Some traditional predictors, such as age, NIHSS score, diabetes, previous stroke, etc., have been proven to be correlated with the prognosis of AIS patients in previous studies (22–24). Consistent with those findings, our results showed that age, NIHSS score and history of stroke were independent predictors of AIS outcomes. Furthermore, we found that PLR levels were associated with prognosis of AIS patients treated with IVT.

Our study suggests that the level of PLR may be a biochemical factor for predicting AIS prognosis. The PLR was calculated by the platelet count/lymphocyte count formula. Numerous studies have shown that both platelets and lymphocytes are predictors of prognosis in ischaemic vascular diseases, especially in myocardial infarction and cerebral infarction (25–28). When AIS occurred, the function of platelets was abnormal (29), and then excessive activation and accumulation of platelets may result in thrombosis and vascular obstruction, further leading to vascular events (30). Stress during acute ischaemic events results in activation of the hypothalamic-pituitary-adrenal axis. As a result, increased cortisol secretion led to a relative reduction in the lymphocyte concentration (31).

It can be speculated that the PLR may provide important extra data for ischaemic events. A number of studies have investigated this point of view. Studies have indicated that PLR is an independent predictor of the incidence and mortality of in-hospital and long-term major adverse cardiovascular events in patients with acute myocardial infarction (32, 33). The PLR levels were higher in patients with poor coronary collateral circulation for stable angina pectoris (34). In addition, Gary et al. (10) proposed that high PLR is significantly associated with patients at high risk for critical limb ischaemia and could be used to highlight patients at high risk for vascular endpoints. In the study of cerebrovascular diseases, an increased PLR was considered a predictor of stroke (35). Studies have suggested that low PLR levels could be a highly negative predictive value to rule out stroke events for patients with carotid artery stenosis (CAS) (36). In another study, high PLR values could indirectly estimate the infarcted size of stoke patients and the poor recanalization rate after thrombectomy therapy (13). Last, an increased PLR has also been shown to play an important role in postoperative stroke after carotid endarterectomy and the development of post-stroke depression (PSD) (37, 38). However, the association between PLR and the prognosis of AIS patients treated with IVT has not yet been studied.

IV rtPA is a key treatment for AIS recommended by the international guidelines (Class I recommendation, Level A evidence) (4, 5, 15, 16). The rtPA not only converts plasminogen to plasmin, which degrades fibrin in thrombus and forms soluble fibrin degradation products (FDPs) but also aggravates platelet activation and aggregation (39, 40). However, considering the risk of intracranial hemorrhage, guidelines generally do not recommend the use of antiplatelets within 24 h of thrombolysis (4, 5, 41). Investigation showed that 14–34% of patients had cerebrovascular reocclusion within 24 h after IVT (42). Urra et al. (43) suggested that lymphopenia is an early feature of stroke, which is a sign of persistent brain damage, stress response, and the greater possibility of infection. In addition, there were reports that lymphopenia after reperfusion in vascular events is an early and efficient predictor of the presence of microvascular occlusion (44). As a result, IVT altered the platelets and lymphocytes in the blood, both of which led to changes in the PLR.

A higher PLR level may indicate revascularization, early neurological deterioration and systemic immune dysfunction, and these events increase the risk of death and unfavorable prognosis in IVT for AIS patients. Therefore, more aggressive thrombectomy or individualized antiplatelet therapy within 24 h after IVT is necessary in patients with high PLR values.

The strengths of this study are that blood samples were obtained within 24 h of symptom onset, meaning that the study was conducted under the ideal conditions to assess the influence of baseline PLR on the outcomes. However, this study has several limitations. First, this was a single-center retrospective study, and the sample size was limited, which may lead to selection bias. Second, although we mainly focused on the relationship between baseline PLR levels and stroke outcomes, perhaps we could obtain more information about this association if PLR levels could be monitored dynamically. Third, we did not completely investigate the difference in PLR between cardiovascular diseases and acute ischaemic stroke.

## Conclusion

Higher PLR levels were associated with an unfavorable outcome and death in AIS patients treated with IVT. PLR might be an inexpensive and effective prognostic factor of AIS patients.

## Data Availability Statement

All datasets generated for this study are included in the article/Supplementary Material.

## Ethics Statement

The studies involving human participants were reviewed and approved by the Ethics Committee of Shanghai Ninth People's Hospital. The patients/participants provided their written informed consent to participate in this study.

## Author Contributions

J-HX conducted the study, collected data, and drafted the manuscript. X-WH participated in the statistical analysis and revised the manuscript. QL, J-RL, M-TZ, and F-FH contributed to the data management and statistical analysis. G-SB designed the study and revised the manuscript. All authors have read and approved the final manuscript.

### Conflict of Interest

The authors declare that the research was conducted in the absence of any commercial or financial relationships that could be construed as a potential conflict of interest.

## Abbreviations

PLR: platelet-to-lymphocyte ratio
AIS: acute ischaemic stroke
IVT: intravenous thrombolysis
rtPA: recombinant tissue plasminogen activator
mRS: modified Rankin Scale
NIHSS: National Institutes of Health Stroke Scale
CT: computerized tomography
MRI: magnetic resonance imaging
CHD: coronary heart disease
AF: Atrial fibrillation
SD: standard deviation
IQR: interquartile range
OR: odds ratio
CI: confidence interval
ROC: receiver operating characteristic
CAS: carotid artery stenosis
PSD: post-stroke depression
FDPs: soluble fibrin degradation products.
