# Supplementary material for: Higher Platelet-to-Lymphocyte Ratio Is Associated With Worse Outcomes After Intravenous Thrombolysis in Acute Ischaemic Stroke
Source: Front Neurol. 2019 Nov 13;10:1192. doi: 10.3389/fneur.2019.01192 (PMC6864121; doi:10.3389/fneur.2019.01192)
Supplement: Supplementary file 1 [file Table_1.docx]

Supplementary Material

**Supplementary Table S1.** Distribution normality by the Kolmogorov–Smirnov test according to the 3-month study endpoints.

|  | Favourable  (n=166) | Unfavourable  (n=120) | Alive  (n=248) | Dead  (n=38) |
| --- | --- | --- | --- | --- |
| Age, years | 0.016 | ＜0.001 | 0.001 | ＜0.001 |
| SBP, mmHg | 0.020 | 0.171 | 0.004 | 0.185 |
| DBP, mmHg | ＜0.001 | ＜0.001 | ＜0.001 | 0.024 |
| TG, mmol/L | ＜0.001 | ＜0.001 | ＜0.001 | ＜0.001 |
| TC, mmol/L | 0.200 | 0.200 | 0.200 | 0.200 |
| LDL, mmol/L | 0.200 | 0.200 | 0.200 | 0.200 |
| HDL, mmol/L | 0.001 | 0.200 | 0.001 | 0.179 |
| HCY, μmol/L | ＜0.001 | ＜0.001 | ＜0.001 | 0.013 |
| Platelet, 10^3/µl | ＜0.001 | 0.002 | ＜0.001 | 0.003 |
| Lymphocyte,  10^3/µl | 0.007 | ＜0.001 | ＜0.001 | 0.071 |
| PLR | ＜0.001 | 0.003 | ＜0.001 | 0.002 |
| NIHSS before IVT, points | ＜0.001 | 0.200 | ＜0.001 | 0.075 |
| Time to needle, min | 0.020 | ＜0.001 | 0.052 | ＜0.001 |

Values are presented as P value for the Kolmogorov–Smirnov test, and for each variable P>0.05 of the two subgroups indicated that the data followed normal distribution.

Abbreviations: SBP systolic blood pressure; DBP diastolic blood pressure; TG, triglycerides; TC, total cholesterol; LDL, low-density lipoproteins; HDL, high-density lipoproteins; HCY, homocysteine; PLR, platelet-to-lymphocyte ratio; NIHSS, National Institutes of Health Stroke Scale; IVT, intravenous thrombolysis.
